# Decreased Sound Tolerance in Tinnitus Patients

**DOI:** 10.3390/life11020087

**Published:** 2021-01-26

**Authors:** Danuta Raj-Koziak, Elżbieta Gos, Justyna Kutyba, Henryk Skarzynski, Piotr H. Skarzynski

**Affiliations:** 1 Tinnitus Department, World Hearing Center, Institute of Physiology and Pathology of Hearing, Mokra 17, 05-830 Warsaw, Poland; d.koziak@ifps.org.pl (D.R.-K.); j.kutyba@ifps.org.pl (J.K.); 2 Teleaudiology and Screening Examination Department, World Hearing Center, Institute of Physiology and Pathology of Hearing, Mokra 17, 05-830 Warsaw, Poland; p.skarzynski@ifps.org.pl; 3 Oto-Rhino-Laryngology Clinic, World Hearing Center, Institute of Physiology and Pathology of Hearing, Mokra 17, 05-830 Warsaw, Poland; skarzynski.henryk@ifps.org.pl

**Keywords:** decreased sound tolerance, hyperacusis, misophonia, tinnitus

## Abstract

(1) Background: Decreased sound tolerance is a significant problem in tinnitus sufferers. The aim of the study was to evaluate the relationship between tinnitus and decreased sound tolerance (hyperacusis and misophonia). (2) Methods: The study sample consisted of 74 patients with tinnitus and decreased sound tolerance. The procedure comprised patient interviews, pure tone audiometry, impedance audiometry, measurement of uncomfortable loudness levels, and administration of the Hyperacusis Questionnaire, Tinnitus Handicap Inventory, and Visual Analogue Scales. (3) Results: The majority (69%) of the patients reported that noise aggravated their tinnitus. The correlation between tinnitus and hyperacusis was found to be statistically significant and positive: *r* = 0.44; *p* < 0.01. The higher the tinnitus severity, the greater the hyperacusis. There was no correlation between misophonia and hyperacusis (*r* = 0.18; *p* > 0.05), or between misophonia and tinnitus (*r* = 0.06; *p* > 0.05). (4) Conclusions: For tinnitus patients the more significant problem was hyperacusis rather than misophonia. The diagnosis and treatment of decreased sound tolerance should take into account not only audiological, but also psychological problems of the patients.

## 1. Introduction

The term “decreased sound tolerance” was introduced by Jastreboff and Jastreboff as a term to cover different types of sound intolerance. Decreased sound tolerance is a disturbance of auditory perception, which may occur as hyperacusis or misophonia [1,2,3]. Hyperacusis is defined as an intolerance to sounds of low to moderate intensity which are not considered loud by most people. The patient’s reaction depends primarily on the physical character of the sound, with the sound’s meaning and context irrelevant [2]. The distinctive feature of hyperacusis is that it appears to be driven by the perceived “loudness” of external sounds [4]. Hyperacusis can have an extremely negative impact on a patient’s life and can prevent them from working and interacting socially. There are numerous descriptions of hyperacusis. Tyler et al. [5] suggest that the clearest way to distinguish different forms of hyperacusis is to focus on loudness, annoyance, fear, and pain. Patients with hyperacusis can experience these reactions singly or in combination. According to Tyler’s distinction, loudness hyperacusis is present when even moderately loud sounds are judged as very loud. Annoyance hyperacusis is a negative reaction to sounds with accompanying avoidance behavior. Fear hyperacusis is defined as another state in which a person is afraid of a particular sound. Pain hyperacusis is present when a patient has a lower pain threshold after exposure to sound [5]. Misophonia proposed by Jastreboff is characterized by emotional reactions such as anger and irritability to specific sounds. Acoustic triggers commonly include human-produced sounds (e.g., munching, chewing, sniffing, audible breathing, whistling) and repetitive sounds that are unnecessarily generated. Negative reactions to sounds are context-specific, so for example the eating sounds made at a family dinner can evoke a negative reaction while the same sounds made at a friend’s house are neutral [2]. There are two distinguishing characteristics of misophonia: presence or anticipation of a specific sound leading to an aversive reaction, and recognition of an unreasonable degree of emotional response (e.g., annoyance, anger, disgust) to the misophonic sound [4]. The reactions are usually limited to, for example, selected family members or colleagues, and there is rather no misophonic reaction if the sound source is a child or animal [6]. Schroder et al. [7] suggested that misophonia might in the future be included in the spectrum of obsessive–compulsive disorders. To measure the severity of misophonia symptoms, they developed an adapted version of the Yale–Brown Obsessive-Compulsive Scale (Y-BOCS), which has been named the Amsterdam Misophonia Scale (A-MISO-S). McKay et al. [8] in their study stated that misophonia is not unequivocally linked to psychopathology, but is a unique set of symptoms. Similarly Rouw and Erfanian [9] did not confirm an increased presence of any particular psychopathology in people with misophonia.

There is little data on the prevalence of decreased sound tolerance. Our own research, conducted in 1999 by a team at the Institute of Physiology and Pathology of Hearing on a group of 10,349 adults, was the first described epidemiological study. Patients were asked to answer the question: Do you have "hypersensitivity" to sounds, pain, or ear discomfort due to sounds?—with the option of a “yes” or “no” answer. Results showed that auditory hypersensitivity was observed in 15.3% of respondents [10,11]. Based on further studies, the prevalence of hyperacusis in the general population was found to range from 6 to 17% [12,13,14].

There is no standard for how to diagnose decreased sound tolerance, and our knowledge of its mechanisms is unproven and speculative. The Hyperacusis Questionnaire has been proposed as a way to assess the extent degree of hyperacusis [15]. The uncomfortable loudness level (ULL) is usually used to assess the presence of hyperacusis [16]. There is also no consensus on testing for ULL. Typically, hyperacusis patients usually show ULL values in the 60–80 dB HL (hearing level) range. In misophonia, both low and normal values of ULL are possible, with a range of 20 to 120 dB HL. Detailed interviews identifying sounds that evoke negative reactions as well as those which are well-tolerated, and properly administered ULL measurements, are crucial for diagnosis of decreased sound tolerance [2].

Decreased sound tolerance is often comorbid with tinnitus, i.e., perception of sound in the absence of an objectively measurable internal or external source. Approximately 15 to 20% of the adult population report tinnitus but only about one in five of those who experience tinnitus seek professional services [17,18]. Clinically important tinnitus is defined as a head or ear noise lasting at least 5 min and occurring more than once a week [19]. Some patients with tinnitus report different forms of decreased sound tolerance. Tyler and Armes were the first to show a relationship between tinnitus and hyperacusis [20]. Jastreboff and Jastreboff [2] estimated that hyperacusis is present in 30% of tinnitus patients and misophonia in 60%. According to Anari [21], 86% of adults with hyperacusis as the primary complaint experience tinnitus as well. On the basis of questionnaires, 17–20% of students experience the negative effects of misophonia in everyday life [22,23]. However, estimates of the association between misophonia and tinnitus are lacking.

The aim of the study was to evaluate the relationship between tinnitus and decreased sound tolerance (hyperacusis and misophonia).

## 2. Materials and Methods

The study comprised patient interviews, audiological evaluation, and administration of self-reported questionnaires. All participants gave informed consent, and the study was performed in accordance with the Declaration of Helsinki. The study protocol was approved by the Ethics Committee of the Institute Physiology and Pathology of Hearing (KB.IFPS.21/2017).

### 2.1. Interview

The interview concerned aspects of decreased sound tolerance (DST) and tinnitus. Patients were asked about the duration of DST, its onset, and the types of sounds causing discomfort. They were also asked if they were afraid of some sounds, if they used hearing protection, and if they avoided particular noisy situations. Patients were asked if noise aggravated their tinnitus and what was the most troublesome problem for them—tinnitus, DST, or hearing loss.

### 2.2. Audiological Evaluation

Audiological evaluation included pure tone audiometry, impedance audiometry, and measurement of uncomfortable loudness level (ULL).

Hearing thresholds were determined for the right and left ears of each patient at frequencies of 0.125, 0.25, 0.5, 1, 2, 4, and 8 kHz (air conduction) and at 0.25, 0.5, 1, 2, and 4 kHz (bone conduction). The results of impedance audiometry were considered abnormal if the middle ear pressure was more negative than −150 mm of H2O and compliance was less than 0.3 cc. The object of the ULL test was to identify the minimum level of sound that was judged to be uncomfortably loud by the subject. The tester gradually made the sound louder and the patient was instructed to press the button (or raise their hand) as soon as the sound became uncomfortably loud. ULL was tested at three frequencies: 1, 2, and 4 kHz. The stimulus was a pure tone.

### 2.3. Questionnaires

Three questionnaires were administered: the Hyperacusis Questionnaire, Visual Analogue Scales, and Tinnitus Handicap Inventory.

#### 2.3.1. Hyperacusis Questionnaire

The Hyperacusis Questionnaire (HQ) was developed by Khalfa et al. [15] to quantify and evaluate various hyperacusis symptoms. It is divided into two parts, but in this study only the second part was used. It comprises 14 self-rated items on three subscales: Attentional, Social, and Emotional. The answers are given on a 4-point scale: “no” (0 points), “yes, a little” (1 point), “yes, quite a lot” (2 points), and “yes, a lot” (3 points). The total score is the sum of the 14 items, with higher scores indicating greater hyperacusis. The authors of HQ proposed that a total score greater than 28 points indicates strong auditory hypersensitivity.

#### 2.3.2. Visual Analogue Scales

Visual Analogue Scales (VAS) were used to evaluate loudness hyperacusis, pain hyperacusis, fear hyperacusis, and misophonia. They consisted of four questions: Are loud sounds uncomfortable for you? Are loud sounds painful for you? Are you afraid of loud sounds? Are human-produced sounds (e.g., while eating, breathing, chewing, sniffing) unpleasant for you? Patients were asked to put a mark on a horizontal line (100 mm long) between its two ends. The left end represented “not at all”, the right end represented “very much”. The VAS score was determined by measuring in millimeters from the left end of the line to the subjects’ marking; the range was from 0 to 100. The higher VAS score, the higher magnitude of loudness hyperacusis, pain hyperacusis, fear hyperacusis, and misophonia.

#### 2.3.3. Tinnitus Handicap Inventory

The Tinnitus Handicap Inventory (THI) was created by Newman, Jacobson, and Spitzer [24] to evaluate the impact of tinnitus on daily living. It is a self-reported measure consisting of 25 items grouped into three subscales: Functional, Emotional, and Catastrophic. For each item there are three possible answers: “yes” (scored as 4 points), “sometimes” (2 points), and “no” (0 points). The responses are summed, with the total THI score ranging from 0 to 100. The higher the score, the greater the perceived tinnitus severity (i.e., the level of distress or impact that tinnitus has on the person; [25]).

### 2.4. Statistical Analysis

Descriptive statistics were calculated for quantitative variables, and percentages were calculated for qualitative variables. The distribution of the HQ and THI global scores was established. One-way analysis of variance (ANOVA) was conducted to compare the levels of hyperacusis and ULL in patients with various levels of tinnitus severity. The relationship between hyperacusis, misophonia, tinnitus, and ULL was evaluated as a Pearson’s bivariate correlation. Statistical significance was established as a *p*-value of <0.05. The analysis was performed using IBM SPSS Statistics, version 24 (IBM, New York, NY, USA).

### 2.5. Participants

There were 74 patients suffering from tinnitus and DST: 35 women and 39 men, aged from 18 to 72 years (M = 44.7; SD = 12.0).

## 3. Results

### 3.1. Audiological Tests

#### 3.1.1. Pure Tone Audiometry

**Figure 1:**
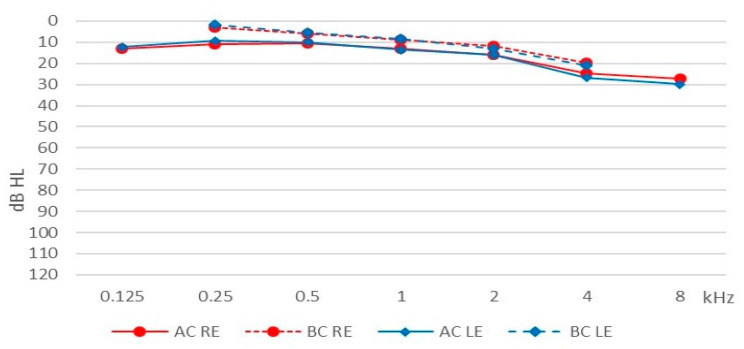
Average hearing thresholds. AC, air conduction; BC, bone conduction; RE, right ear; LE, left ear.

The mean pure tone HL (across all frequencies) for the right ear was 16.60 dB (SD = 12.47) and for the left ear 17.15 dB (SD = 14.49) for air conduction; for bone conduction the comparable figures were 9.78 (SD = 9.64) and 10.12 dB (SD = 10.61). Average hearing thresholds for the patients are shown in Figure 1.

#### 3.1.2. Uncomfortable Loudness Levels 

Table 1 shows data for uncomfortable loudness levels for the patients.

The average ULL (across all frequencies) was *M* = 74.90 (SD = 18.27) for the right ear and *M* = 75.07 (SD = 18.55) for the left.

#### 3.1.3. Impedance Audiometry

Tympanometry was normal (Type A tympanogram) in almost all the patients. Two patients had Type C tympanograms in both ears.

### 3.2. Decreased Sound Tolerance Assessment

#### 3.2.1. Interview

Duration of DST ranged from 0.2 to 40 years (*M* = 7.1; SD = 8.5). There were 29% of the patients who had had DST for less than 1 year, 29% for 1–5 years, 21% for 5–10 years, and 21% over 10 years. The onset of DST was sudden in 46% of the patients and gradual in 54%.

The most often reported types of sounds causing discomfort were: high-pitched sounds for 43% of the patients, loud and impulsive sounds (starting abruptly) for 42%, everyday sounds (traffic noise, household appliances, dishes clanking, etc.) for 35%, and low-frequency sounds for 11%. Only two persons reported that sounds produced by humans (chewing, swallowing, sneezing, etc.) caused them discomfort. 

The majority of the patients (78%) reported that they feared some sounds, 49% used earplugs or earmuffs to reduce noise perception, and 74% tried to avoid some noisy situations (e.g., not attending concerts, not going to the cinema).

#### 3.2.2. Visual Analogue Scales

Results of the VAS measuring hyperacusis loudness, hyperacusis pain, hyperacusis fear, and misophonia are shown in Table 2.

The data indicate that, on average, the patients perceived hyperacusis at a level higher than the middle of the scale. They rated loudness as the highest, but fear was also rated high. Hyperacusis pain had the lowest scoring. The patients scored misophonia lower than hyperacusis.

Three groups of patients were distinguished on the basis of VAS scores for hyperacusis loudness and misophonia: (1) those with predominant hyperacusis; (2) those with predominant misophonia; (3) those with hyperacusis and misophonia at a similar level. The first group consisted of 44 subjects (60%), the second group consisted of 5 subjects (7%), and the third group consisted of 24 subjects (33%).

#### 3.2.3. Hyperacusis Questionnaire 

The frequency distribution of the HQ was examined and is shown in Table 3.

Patients scored highest on items concerning trouble with concentrating and reading in noise, finding noise unpleasant, and noise causing stress. The lowest scores were for items concerning someone else’s opinion about having a problem with noise, using hearing protection, and avoiding or anticipating noisy situations.

**Figure 2:**
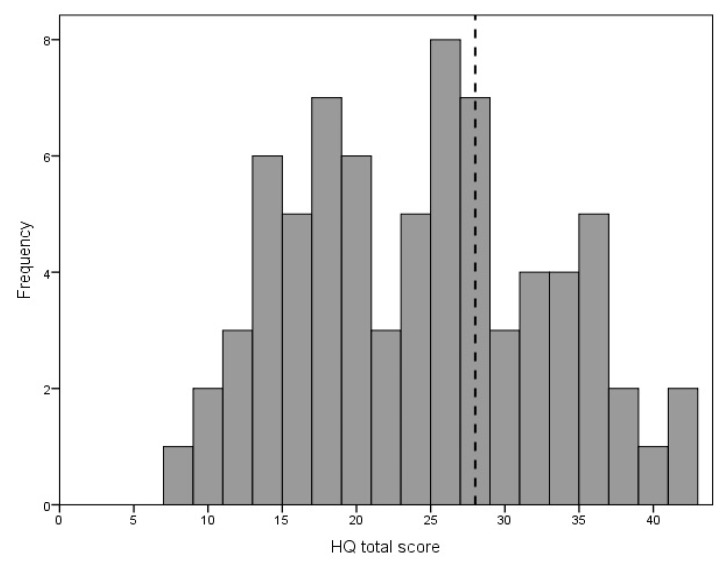
Distribution of Hyperacusis Questionnaire total score. The diagnostic criterion (28 points) is marked with the vertical dotted line.

The mean score for the HQ was 23.76 points (SD = 8.61), range was 8–42 points. The frequency distribution for the total HQ score is given in Figure 2.

Of the 74 participants, 21 (28%) scored above 28 points and, according to the criterion by the HQ authors, could be identified as experiencing strong auditory hypersensitivity.

### 3.3. Tinnitus Assessment

Tinnitus was the most troublesome problem for 66% of the patients, while for 30% it was DST, and for 4% it was hearing loss. The majority (69%) of the patients reported that noise aggravated their tinnitus.

**Figure 3:**
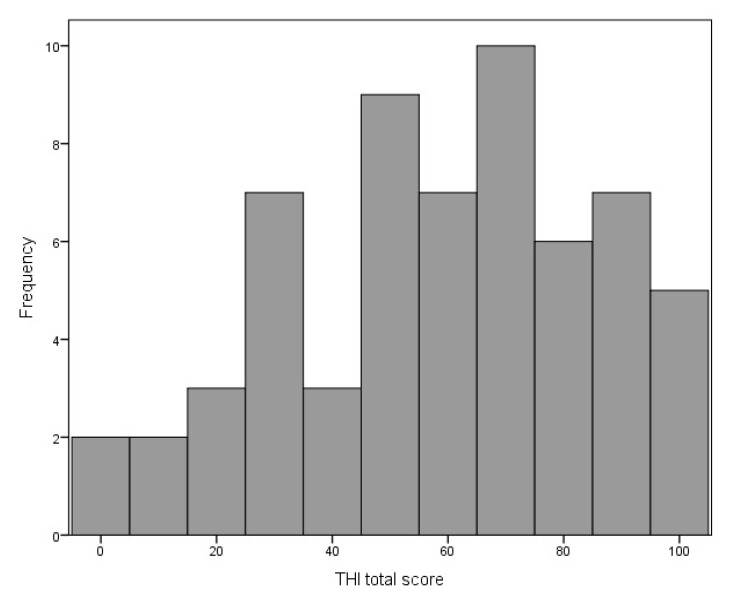
Distribution of the Tinnitus Handicap Inventory total score.

The mean THI score was 58.92 points (SD = 25.83), range was 0–100 points. The distribution of the THI scores is shown in Figure 3.

According to the grading proposed by Skarzynski et al. [26], four groups of patients can be distinguished: those with weak tinnitus (8%), mild tinnitus (23%), strong tinnitus (35%), and very strong tinnitus (34%). Due to the small size, the first group was merged with the second, so that three groups were established: (1) with weak/mild tinnitus; (2) with strong tinnitus; (3) with very strong tinnitus. 

### 3.4. Relationship between Hyperacusis and Tinnitus

**Figure 4:**
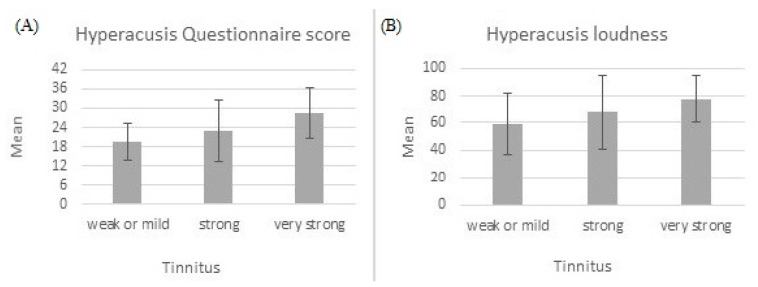
Hyperacusis measured with Hyperacusis Questionnaire (**A**) and hyperacusis loudness measured with Visual Analogue Scale (**B**). Both increase with tinnitus severity.

The above-mentioned three groups of tinnitus patients were compared in terms of hyperacusis, measured both with HQ and VAS. ANOVA results were statistically significant for hyperacusis measured with HQ (*F* = 6.24; *p* = 0.004; *e*^2^ = 0.179) and for hyperacusis loudness (*F* = 3.24; *p* = 0.046; *e*^2^ = 0.102), but statistically insignificant for hyperacusis fear (*F* = 2.51; *p* = 0.091) and hyperacusis pain (*F* = 0.21; *p* = 0.809). There was a significant linear trend for hyperacusis measured with HQ (*F* = 12.10; *p* = 0.001) and for hyperacusis loudness (*F* = 6.46; *p* = 0.014), indicating that as the level of tinnitus severity increased, hyperacusis also increased. Figure 4 shows the trend. 

The three groups of tinnitus patients were also compared in terms of ULL thresholds (the average ULL for the right and left ear across all frequencies was used). ANOVA results were statistically significant both for the right ear (*F* = 3.23; *p* = 0.047; *e*^2^ = 0.111) and for the left ear (*F* = 3.43; *p* = 0.040; *e*^2^ = 0.116). For the right ear, patients with very strong tinnitus (*M* = 68.96; *SD* = 14.41) had significantly lower ULL thresholds than those with weak/mild tinnitus (*M* = 84.44; *SD* = 14.90); however, patients with strong tinnitus (*M* = 73.17; *SD* = 23.56) did not differ significantly from either of the other groups. For the left ear, patients with very strong tinnitus (*M* = 68.33; *SD* = 15.96) had significantly lower ULL thresholds than those with weak/mild tinnitus (*M* = 84.54; *SD* = 12.92), while patients with strong tinnitus (*M* = 73.17; *SD* = 24.07) again did not differ significantly from the other two groups.

Correlations between hyperacusis, misophonia, tinnitus, and uncomfortable loudness level are given in Table 4.

The analysis revealed a relationship between the results of the HQ and VAS scores of hyperacusis. The higher the hyperacusis as measured with HQ, the higher was the loudness hyperacusis, fear hyperacusis, and pain hyperacusis as measured with VAS. Correlations for loudness and fear hyperacusis were moderate; for pain hyperacusis the correlation was weak. 

HQ scores were positively correlated with THI scores, indicating that the higher the hyperacusis, the higher was the tinnitus severity; the correlation was moderate. Hyperacusis as measured with the VAS was also generally related to tinnitus severity, except for hyperacusis pain. For hyperacusis loudness and hyperacusis fear, the correlations were weak. The relationship between hyperacusis pain and tinnitus severity was nearly zero. There was no correlation between misophonia and hyperacusis, or between misophonia and tinnitus.

Correlations between hyperacusis and ULLs were negative, but very weak and statistically nonsignificant. Correlations between misophonia and ULLs were positive, but very weak and statistically nonsignificant as well. The relationship between tinnitus severity and ULL was checked out, and was statistically significant for the right ear. The more severe the tinnitus, the lower the ULL.

## 4. Discussion

Our study sample comprised patients with tinnitus who were admitted to our tertiary referral center due to tinnitus and who had reported problems with decreased sound tolerance. In general, their hearing thresholds were normal (measured with pure tone audiometry) and the results of impedance audiometry were within normal clinical limits.

We found that tinnitus patients with DST had ULL thresholds of approximately 75 dB HL on average. Our findings are in line with those of Anari et al. [21] who found that in a group of 100 patients with hypersensitivity to sounds, the ULL averaged 76.9 dB HL (in that study 86% of the patients also suffered from tinnitus). Slightly different ULLs were shown by Sheldrake et al. [27] in 381 patients with hyperacusis (86% of them also reported tinnitus), where average ULLs were around 85 dB HL. Aazh et al. [28] studied 573 patients attending a tinnitus and hyperacusis clinic and found average ULLs of 85 dB HL. On the basis of results of normal-hearing subjects, Sherlock and Formby (2005) proposed normative ULL values of 100 dB HL at 0.5, 1, 2, and 4 kHz. They said that this limit could be used as a reference to identify subjects who were unusually sensitive to moderate and loud sounds. In comparison to those normative values, we found LDLs in our tinnitus patients to be notably lower. We also found that there was a relationship between tinnitus severity and ULL. Patients with very strong tinnitus had significantly lower ULL thresholds than those with weak/mild tinnitus. Furthermore, Sanchez et al. [29] showed that tinnitus was associated with lower ULL and they suggested three possible underlying mechanisms of that reduced sound level tolerance: 1) apprehension evoked by moderately loud sounds when tinnitus had been experienced; 2) higher auditory attention in tinnitus sufferers; 3) higher stress related to the experience of tinnitus. All three explanations sound reasonable and require empirical verification.

On the HQ questionnaire for hyperacusis, our patients showed elevated scores, with a mean of 23.8 points, a higher score than those found in other studies. In the work of Khalfa et al. [15] the sample consisted of 201 subjects from the general population and their total HQ score averaged 15 points. In the study of Aazh et al. [28] just mentioned, the mean HQ score was 18 points. The study of Fackrell et al. [30] comprised 264 persons with tinnitus and their mean HQ score was 14.9. On this basis, our tinnitus patients appeared to lean quite strongly towards hyperacusis. On the other hand, Khalfa et al. [15] stated that a score greater than 28 represented strong auditory hypersensitivity. The authors did not say how many of their participants scored 28 points or more, but on the basis of the distribution of total scores shown in their work, one can estimate that the rate was about 5%. We think that a criterion of 28 points may be too strong. In our study only 28% of our participants scored above 28 points, while in the interview tinnitus patients said they additionally suffered from DST. Aazh et al. [28] found an inconsistency between HQ scores and ULLs, showing that 95% of the patients with ULLs ≤ 76 dB HL scored HQ levels below the criterion proposed by Khalfa et al. [15]. Aazh et al. [28] therefore proposed a modification of the threshold for diagnosing hyperacusis—a diagnosis of hyperacusis should be based on both ULL and HQ, with cut-offs of ULL ≤ 77 dB HL and HQ score ≥ 22 points. In our opinion this is a reasonable proposition, as it strengthens diagnosis validity, however it does not take into account that the intensity of hyperacusis is a continuum.

In our study, hyperacusis was measured with both HQ and VAS for loudness, fear, and pain. In our previous work [31], evidence was provided that VAS can successfully measure tinnitus severity, and we think that VAS may also be used as a brief screening tool to quickly assess hyperacusis. As Tyler et al. proposed [5], we measured hyperacusis loudness, pain, and fear, dimensions also supported by Schecklmann et al. [32]. We found that loudness and fear were rated rather highly by our patients, higher than was hyperacusis pain. The VAS scores correlated with the HQ scores (loudness 0.52; pain 0.36; fear 0.58), but the correlations were moderate or weak. If higher correlations could be found, they would fully confirm the validity of VAS.

Tinnitus severity in our study was quite strong. Weak or mild tinnitus was revealed in 31% of the participants, while 69% showed strong or very strong tinnitus. The mean score for the THI was 58.9 points; in comparison, Aazh et al. reported an average value of 44.7 points for the THI [28], and Fackrell et al. 35.0 points for the THI [30].

In our study, the correlation between hyperacusis and tinnitus severity (the HQ and THI scores) was *r* = 0.44, similar to that obtained by Fackrell et al. [30] (*r* = 0.49). Gilles et al. [33] examined 588 patients who visited a hospital with tinnitus as the primary complaint. The correlation between tinnitus severity (as measured with the Tinnitus Questionnaire) and hyperacusis (measured with the HQ) was *r* = 0.5. The authors concluded that the presence of hyperacusis may intensify the severity of perceived tinnitus. Cederroth et al. [34] also showed that hyperacusis is strongly associated with tinnitus, and the relationship increased with tinnitus severity. We have provided evidence that subjects with weak/mild, strong, or very strong tinnitus differ in terms of how they perceive loud sounds—the more severe the tinnitus, the more severe is the hyperacusis.

We found that 69% of tinnitus patients reported that their tinnitus was made worse by noise. This is in line with the findings of Schecklmann et al. [32] that tinnitus patients with hyperacusis had a higher probability (82%) that their tinnitus was affected by external sound and noise, in contrast to non-hyperacusis tinnitus patients (42%). They concluded that hypersensitivity to sound may be a clinical criterion for a distinct subtype of tinnitus. They also found that tinnitus patients with comorbid hyperacusis scored higher on tinnitus and depression questionnaires [32].

Hyperacusis patients are usually recommended sound therapy to desensitize their condition—exposure to gradually increasing sound levels from apps or sound generators. Sound therapy appears to be beneficial for either hyperacusis alone or hyperacusis coexisting with tinnitus. An indication from the patient about whether tinnitus or hyperacusis is their major complaint is important because it determines further treatment. In our hyperacusis patients we usually start therapy with a soft sound that is accepted by the patient and gradually increase its intensity. Patients with severe tinnitus and hyperacusis may also suffer from anxiety and depression disorders and are always referred for psychological therapy. In the case of suspected depression, they are referred to a psychiatrist.

Decreased sound tolerance comprises not only hyperacusis, but also misophonia. Little is known about the extent of misophonia in tinnitus sufferers. Jastreboff and Jastreboff [35] found that of 149 patients attending a tinnitus and hyperacusis clinic, 29% were diagnosed with pure misophonia (without hyperacusis) and 28% had both misophonia and hyperacusis. In total, 57% of tinnitus patients had misophonia. Observations from our clinical practice and this study’s results fail to confirm that misophonia is prevalent among tinnitus patients.

Our study indicates that misophonia is generally not a frequent problem for tinnitus patients. Only two persons said in the interview that human-produced sounds cause them discomfort. On the VAS, patients scored misophonia to be 39 points on average, while scores for hyperacusis loudness were much higher (on average 70). Misophonia as measured with the VAS did not correlate with hyperacusis or with that measured with the HQ; similarly, it did not correlate with VAS measures of hyperacusis loudness, pain, or fear. The novel feature of these results is that we have shown in a group of tinnitus patients that misophonia is a different symptom from hyperacusis—misophonia relates only to specific sounds, while hyperacusis relates to all sounds above a certain intensity. At our institute, patients with suspected misophonia are routinely referred to a psychologist and sometimes for psychiatric consultations.

Because there are no uniform diagnostic or therapeutic criteria for hyperacusis or misophonia, it is important to inform the patient that knowledge about reduced tolerance to sound is currently incomplete and therefore that any proposed therapy will have limited effectiveness.

## 5. Conclusions

Decreased sound tolerance is a significant problem in tinnitus sufferers. The diagnosis of decreased sound tolerance should be a multi-specialist process involving audiologists, psychologists, and psychiatrists.

## Figures and Tables

**Table 1:** Uncomfortable loudness levels (dB HL).

	Hz	*Min*	*Max*	*M*	*SD*
	1000	20	105	74.04	18.55
Right ear	2000	25	110	73.75	18.25
	4000	25	120	76.91	19.83
	1000	25	120	74.85	18.87
Left ear	2000	20	115	73.82	18.55
	4000	25	120	76.54	19.67

Min, minimum; Max, maximum; M, mean; SD, standard deviation.

**Table 2:** Results of Visual Analogue Scales (VAS) measuring hyperacusis (loudness, fear, pain) and misophonia.

VAS	*Min*	*Max*	*M*	*SD*
Loudness	10	100	69.99	23.11
Pain	0	100	47.59	32.43
Fear	4	100	64.00	28.79
Misophonia	0	100	38.55	34.28

Min, minimum; Max, maximum; M, mean; SD, standard deviation.

**Table 3:** Frequency distribution of Hyperacusis Questionnaire responses.

Item	Item Content	Frequency of Responses for Items (%)				*M*	*SD*
		0	1	2	3		
1	Use earplugs/earmuffs to reduce noise perception	28.8	37.0	12.3	21.9	1.27	1.11
2	Harder to ignore sounds	11.0	39.7	28.8	20.5	1.59	0.94
3	Trouble reading in noise	4.1	25.7	27.0	43.2	2.09	0.92
4	Trouble concentrating in noise	2.7	18.9	37.8	40.5	2.16	0.83
5	Difficulty listening to conversation in noise	6.8	23.3	30.1	39.7	2.03	0.96
6	Has anyone told you that you tolerate noise badly	31.5	41.1	12.3	15.1	1.11	1.02
7	Particularly sensitive to street noise	16.2	32.4	21.6	29.7	1.65	1.08
8	Noise unpleasant in certain situations	6.8	21.6	23.0	48.6	2.14	0.98
9	Anticipate noise before going out	27.0	31.1	10.8	31.1	1.46	1.20
10	Turn down invitation because of noise	27.0	44.6	9.5	18.9	1.20	1.05
11	Noise bothers more in a quiet place	7.2	46.4	23.2	23.2	1.62	0.93
12	Stress reduces ability to concentrate in noise	10.8	28.4	27.0	33.8	1.84	1.02
13	Less able to concentrate in noise at end of the day	12.2	37.8	20.3	29.7	1.68	1.04
14	Noise causes stress	2.7	28.4	24.3	44.6	2.11	0.92

M, mean; SD, standard deviation.

**Table 4:** Pearson’s correlation coefficients between hyperacusis, misophonia, tinnitus, and ULL.

	VAS-L	VAS-P	VAS-F	VAS-M	THI	ULL RE	ULL LE
HQ	0.52 **	0.36 **	0.58 **	0.18	0.44 **	−0.13	−0.16
VAS-L		0.37 **	0.57 **	0.09	0.34 **	−0.06	−0.06
VAS-P			0.39 **	−0.06	0.04	−0.04	−0.02
VAS-F				0.18	0.37 **	0.06	0.04
VAS-M					0.06	0.10	0.09
THI						−0.24	−0.28 *
ULL RE							0.81 **

** *p* < 0.01; * *p* < 0.05. HQ, Hyperacusis Questionnaire; L, loudness; P, pain; F, fear; M, misophonia; THI, Tinnitus Handicap Inventory; ULL, uncomfortable loudness level; RE, right ear; LE, left ear.
